# Proposed Amendment of the Truck Acts, 1831 to 1896, and How It May Affect the Voluntary Hospitals

**Published:** 1914-07-04

**Authors:** William Straker

**Affiliations:** Corresponding Secretary Northumberland Miners' Association, Member of the House Committee of the Royal Victoria Infirmary


### PROPOSED AMENDMENT OF THE TRUCK ACTS, 1831 to 1896, AND HOW IT MAY AFFECT THE VOLUNTARY HOSPITALS.

**By WILLIAM STRAKER, Corresponding Secretary Northumberland Miners' Association, Member of the House Committee of the Royal Victoria Infirmary.**

Sub-section (*c*) of Section 1 combined with the section reads as follows: "An employer shall not make any deduction or require any payment in respect of" "any subscription or payment to, or otherwise in connection with, any benefit society, sick club, hospital, or any other society, club, or benefit, or for medical attendance."

I want to say at once that, in my opinion, the passing of the sub-section into law will injuriously affect voluntary hospitals, and, therefore, in the interest of the working classes, ought to be opposed.

Because I suggest such opposition, it must not be understood that I am against the Bill as a whole. I recognise the necessity of the Truck Acts being amended in order to prevent the continuation of many hardships to the workers, especially in our textile industries.

I am against this sub-section because it is not a necessary part of such a Bill, and will, if retained in the measure, stop a form of deduction from wages which the workers have freely and intelligently requested employers to make, in order that they and theirs may receive, when needed, in hospitals the benefit of that up-to-date and expert medical and surgical skill which otherwise they could not receive.

We may be told that this difficulty can be got over by nationalising the hospitals. With that view I am strongly in sympathy, holding, as I do, that, in the future, State hospital provision will be regarded as a necessary equipment of a civilised people.

The growing difficulty of providing adequate hospital accommodation for those who cannot afford the expense of treatment in private hospitals is such that the eyes of many people are turning toward State assistance or provision as the way out of the difficulty.

But "while the grass is growing the horse is starving," and so I am against anything, such as this sub-section, which would at one blow almost paralyse the present voluntary hospital system without first putting anything else in its place.

The causes which led to the passing of the first Acts of Parliament for the prevention of paying wages other than in the coin of the realm throw a lurid light on the treatment of the working classes by employers of labour; and the necessity of an amendment and extension of the Truck Acts reflects no credit on our captains of industry of the present day.

While vested interests have always stood in the way of the passing of long-overdue legislation, yet, on the other hand, wise statesmanship consists in only bringing from time to time the laws of the country abreast of the ever advancing moral and intellectual status of the people; and so when the moral conscience of the people had sufficiently developed as to be violated by the legal robbery of the workers in some of our large industries, these Acts were passed.

The beginning of the history of this sort of legislation is almost lost in the musty records of the past. In the year 1464, during the reign of Edward IV., an Act was passed which applied only to woollen clothworkers, and enacted that "*carders, spinsters, and all such other labourers, in any part of the said trade*" shall be paid "*lawful money for all their lawful wages and payment of the same.*"

In 1725, when George the First was king, weavers were attempting to form trade unions for self-protection, and an Act was passed providing heavy penalties for such combinations, but it also provided that the employer had to pay "*the full wages or other price agreed on in good and lawful money of the Kingdom.*"

This latter provision was, in 1749, in the reign of George II., extended to many other industries, and in 1817, in George III.'s reign, it was extended to the coal-mining industry.

There are many ways of killing a dog without hanging it, and many ways of getting through an Act of Parliament without violating the letter of it. This keeping of the letter while violating the spirit led, among other things, to a miners' strike in Northumberland and Durham in the year 1831. On March 21 of that year the miners of these counties held a mass meeting on Newcastle Town Moor, and decided to come out on strike if their grievances were not remedied. Among the resolutions passed at that meeting was the following:

"That no man in future buy meat, drink, or candles from anyone connected with the collieries."

In Fynes' "History of the Miners of Northumberland and Durham" we read: "This last resolution was intended to put a stop to the existence of those establishments known as 'Tommy shops,' a system by which a miner and his family were placed completely at the mercy of the colliery owners. The 'Tommy shop' was generally kept by a relative of the viewer of the colliery, the pitman was compelled to purchase his provisions there, and his wages were confiscated at the pay-day to settle any balance there might be due to the 'Tommy shop' keeper."

As the Truck Act of 1831 was passed on October 15 of that year, the pitmen of Northumberland and Durham may fairly claim to have played an important part in the passing of the measure.

Section 2 of that Act made it illegal to place in any contract between employer and employed, in trades enumerated in the Act, any stipulations as to the manner in which the wages were to be expended. This put an end to the "Tommy shop," but a system of robbery by fines went on.

In the year 1843 the coal-hewers of Thornley, in Durham, refused to work under a system of fines at that colliery, and in consequence were taken to Court, and, notwithstanding the terrible injustice of which they were victims, as revealed by the evidence at the Court, they were condemned to six months' imprisonment. The case was taken to the Court of Queen's Bench, and, happily, owing to some informality, they were acquitted.

The fines complained of were for sending to the surface one quart of foul or splint coal in a 6-cwt. tub of good coal. Owing to the dim light of the miner's lamp it was impossible to prevent this foul coal from being filled along with the good coal, as it constituted part of the coal seam.

In many cases after men had worked a fortnight their earnings were confiscated by these fines, with the exception of 3s. or 4s. In one case brought before the Court a hewer was fined 12s. for three days, and in consequence stood 3s. in debt to the colliery owner.

Bradlaugh's Truck Act of 1887 strengthened and extended the Act of 1831, but did not make fines illegal, such as those which caused the strike at Thornley. Also, as the 1831 Act had in many cases been ignored by employers owing to no one being charged with the responsibility of seeing that it was observed, the 1887 Act empowered factory and mines inspectors to take action against employers guilty of any infringement of the Act.

There was nothing, however, in the measure to prevent the continuation of fines, but among miners in many districts where they had a good trade union the worst form of the evil was destroyed; it was, however, not until the Truck Act of 1896 that the law stepped in and, under certain conditions, made fines illegal. The inadequacy of that Act to prevent the evil arising from systems of deductions from wages, especially among female workers where trade unionism is weak, has made the present amending Bill necessary. The promoters of this Bill, feeling indignant because of the grossly unjust way in which employers have made, in spite of their workers, deductions from wages, have proposed the sub-section we are against in order to disallow any deductions whatever. While we cannot help sympathising with them and sharing their indignation, yet, after the most careful consideration of the probable effects of the proposal, we must conclude that it is indicative of more feeling than sound judgment.

On April 5, 1906, the Rt. Hon. H. J. Gladstone, M.P., then Secretary of State for the Home Department, appointed a Departmental Committee on the Truck Acts. Their inquiry lasted for two years and eight months. They held eighty-two meetings and examined 118 witnesses. In December 1908 their Report was issued.

For the purpose of this paper it is only necessary to look at that part which deals with deductions from wages for hospitals.

Among the witnesses examined were Mr. D. Shackleton, now holding an appointment as a permanent official under the Government in connection with the National Insurance Commissioners; Mr. B. Turner, representative of the workers in the woollen trade, and at the present time Mayor of Batley; Mr. Gavin, representing the Amalgamated Union of Iron and Steel Workers of Great Britain; and Mr. R. Smillie, who is now president of the Miners' Federation of Great Britain. These men favoured the system of making deductions from wages for hospitals, Mr. Smillie expressing the opinion that it is "a very fine thing that workmen should so contribute." They did, however, hold that there should be no compulsion on workmen to do so.

Miss Black, of the Women's Industrial Council, said there were places where the expectation was so great that every worker would contribute that it was practically compulsory.

Mr. Cadbury was the only employer giving evidence who made compulsory contributions for this purpose, while he was strongly against fines of any kind.

Representatives of hospitals thought that the hospital funds would suffer seriously if this method of collection was stopped and contributions were left to the men to make individually, because of the lack of organisation. They also said that the workmen were well represented on the various boards of management of the hospitals.

The Majority Report of the Departmental Committee on this matter is as follows:—

"We think it may be inferred from the evidence that cases of undue pressure are few, and it does not appear to us that any interference with the system is called for. We recommend that deductions from wages for contributions to hospitals should be allowed."

In their "Summary of Recommendations," No. 28 reads:—

"That in respect of hospitals there should be no alteration of the existing law."

In my opinion there is a lot of cant-talk against compulsory payments for this and other purposes. If it is true, as Miss Black stated, that every worker was so strongly expected to contribute that it was practically compulsory, it is the greatest compliment to the unseen influence of human goodness. If right were not to continue stronger than wrong; if goodness were to cease to be more powerful than evil, wrong and evil would triumph, and all humanitarian institutions, such as hospitals for the sick and lame poor, would perish.

While I would not advocate the right of any employer of labour to make it compulsory for his workpeople to contribute to anything of the kind, yet I am absolutely convinced that there are a number of people who must be compelled to do their duty and contribute to a common fund for a common purpose for their own good and the good of society, otherwise civilised society could not exist. Where there is a community of people with common needs and common interests, the rule of the majority must prevail; it is "the only way" by which such a community can protect itself against those who would take advantage of being units in that community without sharing the common burden.

All rates and taxes are based on this principle, and also contributions under our National Insurance Act. I know there are people who would escape payment of all rates and taxes if they could, and there are also people who object to contributing to the National Insurance funds, and there are others who for their own purposes endeavour to persuade the people that such contributions are wrong and violate the so-called "sacred right of the individual." Such people as these are either too selfish or too blind to understand the requirements of a complex human society such as we have in all the great civilised countries of the world.

I want now to refer to the Minority Report of the Departmental Committee, signed by Mrs. H. J. Tennant, ex-superintending woman inspector of factories, and Mr. Stephen Walsh, M.P. (Mr. Walsh has his name on the back of the Bill we are now dealing with.)

This report commences as follows: "Sir,—It is with deep regret we find ourselves unable to sign the report. We are in cordial agreement with certain of the recommendations (viz., 2, 3, 4, 15, 16, 20, 22 (a), 25, and 27), so far as they go, but we are unable to agree with the three chief proposals, which would maintain—

"(1) The system of fines.

"(2) The system known as the living-in system. From for bad work and injury to material and other property; and

"(3) The system known as the living-in system. From these three systems we would withhold the sanction of the law.

"Also we are unable to concur in certain of the minor recommendations. We are in disagreement with Recommendations 12, 17, 18, 19, 21, 22 (d), 23, and 24, which deal with the bonus system, houses, benefit societies, messrooms, medical attendance, the supply of tools, food and fuel, and with service rendered by the employer in relation to work done by the workman."

Neither here nor in their "Summary of Conclusions" do they refer to No. 28 of the Majority Report recommendations, which suggests that so far as deductions for hospitals are concerned the law should remain unaltered.

I have traced the history of the Truck Acts, 1831 to 1896, and the findings of both reports of the Departmental Committee in order to show that nowhere has there been a case made out for the prohibition of deductions from wages, made at the wish of the workers themselves, of contributions to hospitals.

The old evils and the evils which the promoters of this Bill want to abolish were, and are, conditions against which the workers protested and are protesting. But the system by which workmen so largely contribute to hospitals is not a condition of hiring, and therefore ought to be outside the prohibitions of any Act of Parliament.

The National Anti-Sweating League has issued a circular on the Truck Acts Amendment Bill. This circular specifies the evils which need to be remedied, but deductions for hospitals are not one of them. It does, however, advise the prohibition of all deductions so as to get completely clear of the deductions and fines complained of. This, too, seems to be the idea of the supporters of Sub-section (e) of Section 1 of the Bill.

To me there appears in this little statesmanship, if any. It certainly would be effective with that effectiveness which one would expect to see in the case of a gardener who might be so foolish as to uproot the whole of his crops in order to get clear of the weeds.

Probably nowhere in the world is there a larger subscription by working-men than that in connection with the Royal Victoria Infirmary of Newcastle-on-Tyne. For the year 1913 it amounted to over £20,000. Experience has shown us that it would be impossible to get at the most more than 50 per cent. of this by any other means than by deductions from wages at the pay offices.

Since the passing of the 1911 Coal Mines Act, the workmen at a large number of our pits in Northumberland have exercised the right given them by this Act and claimed to be paid weekly instead of fortnightly as previously. In consequence of the greater amount of office work entailed by this change, a few colliery owners have refused to make the deductions for the infirmary and other charitable purposes. The workmen have sent a number of deputations to the managers of these pits begging of them to recommence making these deductions. In some cases the managers still refuse, and the consequence is that not one fourth of the usual contributions has been sent to the Infirmary from these pits. This is not because workmen do not want to contribute, but because of the lack of that organisation which is necessary for the success of any business. Other forms of organisation for this purpose have been tried, but with little success. Surely there ought to be better reasons given than have yet been advanced before we stop, by a legal enactment, working-men doing their own business in their own business way.

At the present time our Infirmary waiting list has over 1,300 names on it, owing to the lack of accommodation. If our income were to be reduced in the way it would be in case this Bill is passed into law with this sub-section intact, we would be driven, for lack of money, to either close some of the already too few wards or to make a charge for admission to the hospital. In either case large numbers of suffering poor people, who otherwise would receive the benefits of the institution, would have to die for lack of that treatment which they cannot afford to get any other way than in a free hospital such as the Newcastle Infirmary.

Newcastle-on-Tyne is in the centre of a coal-mining district, and about one in every four beds in the Infirmary is occupied by a miner or a member of a miner's family Every day we have men from our mines, and from other of our great Tyneside industries, carried in. During the year there are hundreds of such. To do anything that would hinder them from being admitted would be even a greater calamity, though not so conspicuous, as any of the great pit explosions we have had during recent years.

In face of circumstances such as these, which are not peculiar to Newcastle-on-Tyne, it is to be hoped that when this Bill is again brought forward, or when a Government measure is brought in to amend the Truck Acts, which is probable, this sub-section will be either deleted or modified in such a way as to permit of workmen's contributions to hospitals being deducted from wages at the pay office, so that not only the wealthy, but the poor also, may have all the benefit of the marvellous surgical and medical discovery and expert skill which is the glory of the twentieth century and probably the greatest triumph of all the ages.

### THE DISCUSSION ON MR. STRAKER'S PAPER.

The Chairman moved, and Dr. Mackintosh seconded, a vote of thanks to Mr. Straker for his instructive paper, which was cordially carried.

### The Earl of Winterton said that he was in favour of the expressions embodied in the paper as to the Amending Bill in connection with the Truck Acts, which had now been dropped. He suggested that, as the matter was a very important one, if the measure dealing with the Acts was brought forward this session, Mr. Straker might undertake to confer with the Labour Party, and get them to bring forward an appropriate amendment, and he (Lord Winterton) would undertake, if he were allowed to see what was agreed upon by the Labour Party, to approach his colleagues who were connected with hospital management on both sides of the House, and get them to agree to the amendment also.

Mr. Straker, in reply, said that Lord Winterton and all of them could rest assured that such a measure would be brought before the notice of the Labour Party, and before that it would be brought before the Miners' Federation of Great Britain, of which the miners of Northumberland formed a part, and he happened to be on the committee of this Federation. Miners had a much wider interest in this sub-section than what was covered by the term "hospitals." Generally throughout Great Britain miners had what were called "doctors' clubs" for the provision of medical treatment other than for insured members of the family. These clubs would also be destroyed if the sub-section were carried.

The Chairman moved the following resolution: "That this meeting of the British Hospitals Association representing the voluntary hospitals in general of the United Kingdom are of opinion that any legislation prohibiting deductions from wages by way of subscriptions to hospitals will result in contributions from employees in a great number of instances not being paid, and will thus impair the efficiency of many institutions at present conferring invaluable benefits on industrious and deserving workpeople. They therefore hope that in any amendment of the Truck Acts the interests of the voluntary hospitals and kindred institutions will be safeguarded."

Dr. Mackintosh seconded the motion, which was carried *nem. con.*, and it was arranged that a copy should be forwarded to the Home Secretary.

### THE BUSINESS MEETING.

After the proceedings of the Conference had been concluded a meeting was held for the purpose of electing the officers for the ensuing year.

Dr. Mackintosh moved a cordial vote of thanks to the committee of management of the Newcastle Royal Infirmary and to the house governor, Mr. R. H. P. Orde, and his staff for the excellent arrangements made for the Conference. He specially acknowledged the services of Mr. Orde as the honorary local secretary for the Conference. The resolution was seconded and carried.

Mr. Conrad W. Thies moved a vote of thanks to the Lord Mayor of Newcastle and to the City Council for their civic welcome, which was heartily accorded. Dr. Mackintosh also moved a hearty vote of thanks to Councillor W. J. Sanderson for the admirable way in which he had conducted their proceedings as chairman. This motion was also carried. On the motion of Mr. Alexander Hayes a vote of thanks was passed to the contributors of the papers.

### The New President.

On the motion of the Chairman, Councillor Johnstone Wallace, J.P., Lord Mayor of Newcastle, was unanimously elected as the President of the Association for the year; and, on the motion of Mr. Howard Collins, Sir Henry C. Burdett, K.C.B., K.C.V.O., Sir William Cobbett, and Sir William Osler, Bart., M.D., were elected Vice-Presidents.

Mr. Howard Collins said that at a meeting of the Council on the previous night Dr. Mackintosh had intimated that he must withdraw from the chairmanship of the Council. They all knew the useful service that that gentleman had rendered to them, and at the Council meeting a very cordial vote of thanks was passed to him for the work that he had done on behalf of the Association during the past five years, first as hon. secretary and later as Chairman of the Council. He moved that Dr. Mackintosh be elected a Vice-President of the Association in recognition of his valuable service.

Sir Henry Burdett, in seconding the resolution, said they were under a deep debt of gratitude to Dr. Mackintosh for his labours, and he sincerely hoped that the resolution which had been passed by the Council would be engrossed on vellum and presented at the next annual meeting to Dr. Mackintosh.

The motion was warmly carried.

Dr. Mackintosh, in responding, said he could assure them that, though he was leaving the chair, his interest in the work of the Association would not cease, and that he would continue to do his best, in his humble way, for their very useful organisation. He hoped to be able to attend their annual meetings, and he hoped also that the Association would go on and prosper. A good deal of spade-work had already been done. He valued very highly the honour they had done him in electing him a Vice-President.

Mr. Stewart Johnson and Mr. R. A. Owthwaite were re-elected as Hon. Treasurers, Mr. Conrad W. Thies and Mr. Alexander Hayes as Hon. Secretaries, and Mr. Arthur F. Whinney as Hon. Auditor, all these gentlemen being thanked for their past services.

The members of the Council were re-elected, and the following were appointed to fill vacancies on that body, on the motion of Mr. Stewart Johnson: Earl Winterton, M.P., Sir William Bull, M.P., Dr. W. G. Hume, Newcastle; Mr. P. J. Michelli, Greenwich; Mr. Roden H. P. Orde, Newcastle; and Mr. C. S. Risbec.

### The Next Conference.

It was agreed to accept the invitation to hold the next Conference in Liverpool, and it was suggested by Sir Henry Burdett that in future the Conferences should be held at a later period in the year, say September or October.

At the close of the proceedings a meeting was held of the newly-elected Council, when Mr. J. Wade Deacon, Chairman of the Royal Infirmary, Liverpool, was unanimously elected Chairman of the Council for the ensuing year. An executive committee was also appointed.

